# Supplementary material for: Erdheim–Chester Disease: Investigating the Correlation between Targeted Treatment Therapy and Disease Outcomes
Source: Cancers (Basel). 2024 Mar 27;16(7):1299. doi: 10.3390/cancers16071299 (PMC11010843; doi:10.3390/cancers16071299)
Supplement: Supplementary file 1 [file cancers-16-01299-s001.zip › cancers-2855826-supplementary.pdf]

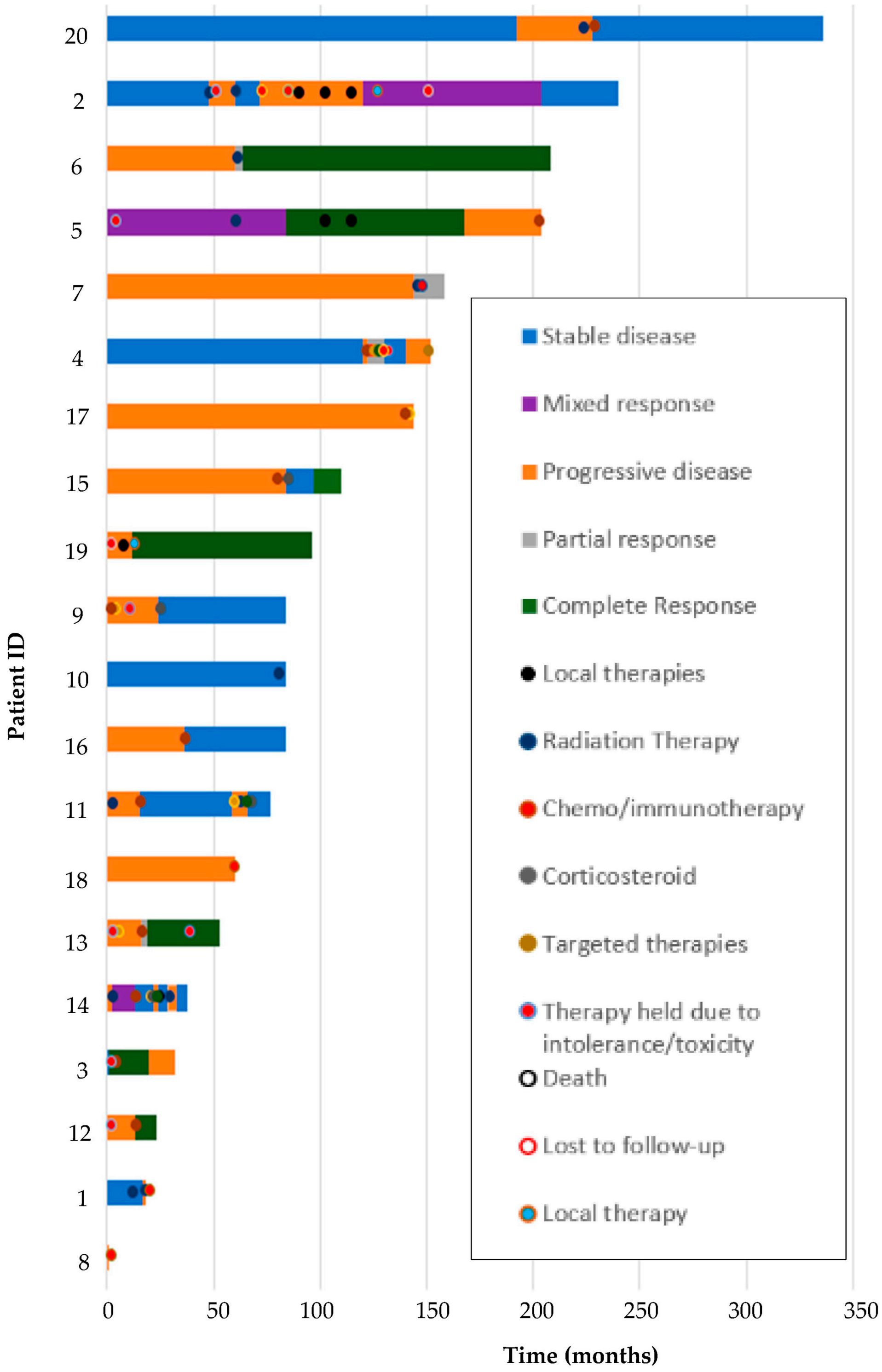

Supplemental Figure S1. Swimmer's plot for all 20 evaluated patients. Different colors designate each type of response (complete, partial, progressive, and mixed) for visualization of data with respect to therapy during treatment course. All treatments (indicated by a different colored circle) were included as follows: local therapies (intralesional injections and surgery), radiation, corticosteroids, chemo and immunotherapy (referring to any chemotherapeutic/immunomodulatory agent or monoclonal antibody), corticosteroids, and targeted agents (referring only to small molecule inhibitors of Ras/MAP kinase signaling). Additional outcomes visualized on the plot included treatment discontinuation due to adverse effects/intolerance and loss of patients to follow-up or death.

Supplemental Table S1: Demographics, diagnostics, and treatment responses for patients within this cohort.

Patient ID	Biologic Sex	Age at Diagnosis	Disease by imaging?	Imaging modality	Primary anatomic site	Additional disease sites	Molecular diagnostics utilized?	If so, which modality (IHC, molecular)?	Additional IHC or molecular testing, with specific description, if applicable	Treatment summary	Response assessment	Best Response	Highest monocytes in the absence of therapy or prior to/in the course of disease progression (N/A if no disease progression or no data available)	Monocytes with re-institution of therapy (N/A if no disease progression or data not available)
1	Male	64	Yes	PET	Testicles	Axial and appendicular skeleton, retroperitoneal fibrosis, mediastinum (Pericardial effusion)	Yes	IHC	CD68 and S100 positive. CD45 with mixed inflammatory infiltrate and emperipolesis.	Diagnostic workup/observation 12 months -> PEG-Interferon (5 months, progressive disease) -> Cladribine (unknown duration, lost to f/u as patient following with local Hematologist).	Clinical, PERCIST	Unknown	0.4, 8.3%	N/A (no data available)
2	Male	15	Yes	MRI, CT	Eyelid, Pituitary	Pons, pituitary (hypogonadism, diabetes insipidus), bilateral hips (back pain), nasal bone	Yes	IHC	Juvenile xanthogranuloma on initial path. Several biopsies since then supporting ECD diagnosis. BRAF negative.	Observation (36 months, stable disease) -> Intralesional corticosteroids of corneal lesions, PO steroid, vinblastine (12 months, progressive disease) -> Observation (12 months, stable disease) -> Cladribine x 2 cycles (unknown response) -> Rituximab (1-month, unknown response) -> PEG-Interferon (12 months, unknown response) -> Corneal transplant, multiple surgical resections of eyelid lesions, thalidomide, lenalidomide, radiation and clofarabine (over 7 years from 2012 to 2019, mixed response) -> Observation (2019–2021, stable disease).	Clinical, RECIST	Stable disease	0.7, 24%	0.4, 7.2%
3	Male	75	Yes	PET, CT	Lung	Diffuse axial bone involvement, mediastinal lymphadenopathy	Yes	IHC, NGS	CD 163 and S100 positive. BRAF V600E staining negative. NGS positive for BRAF V600E mutation.	Trial of high dose steroids (1-month, stable disease) -> Dabrafenib (19 months, complete metabolic response) -> New progressive disease (12 months), continuing dabrafenib.	Clinical, PERCIST, RECIST	Complete metabolic response	0.8, 12.4%	N/A (Remained on same therapy)
4	Female	14	Yes	PET, CT, MRI	Craniofacial	Hepatomegaly, splenomegaly, perinephric soft tissues, paravertebral, hemopericardium, osseous lesions, mediastinum, pericardium, lungs, cervical/spinal and meningeal involvement	Yes	IHC, NGS	CD 163 positive. Mutational testing for V600E BRAF mutation and ALK rearrangement negative. MiOncSeq with t(1;7) RNF11-BRAF fusion, somatic diploid GNAS amplification, WT1 and WNT2 positive. No germline mutations.	Treated with bisphosphonate for 10 years with presumed diagnosis of fibrous dysplasia -> Progressive multifocal disease, started Cobimetinib (2 months, partial response. stopped due to intolerance) -> Binimetinib (2 months, partial response, stopped due to intolerance) -> PEG-Interferon (2 months, unknown response, stopped due to intolerance) -> Off therapy (12 months, progressive disease) -> Trametinib started, continuing on therapy. Unknown disease response to date.	Clinical, PERCIST, RECIST	Partial metabolic response	0.9. 9.7%	0.5, 8%
5	Female	39	Yes	PET	Ocular, periorbital swelling	Diffuse lymphadenopathy, shoulder subcutaneous soft tissue nodularity	Yes	IHC	Follicular lymphoid hyperplasia with focal progressive transformation of germinal centers, polytypic plasmacytosis, predominance of IgG4-positive plasma cells	Several doses of intralesional steroids into periorbital and ocular lesions (84 months, mixed response, immediate improvement but then recurrence) -> Rituximab (2 months, subjective improvement in periorbital swelling, but no imaging to assess response) -> Multiple debulking surgeries and intraocular steroid injections (84 months, complete metabolic response) -> New lesions, started Cobimetinib (unknown response).	Clinical, PERCIST	Complete metabolic response	0.7, 10%	0.6, 9.4%
6	Female	66	Yes	CT, MRI	Diffuse lymphadenopathy	Lytic bone lesions	Yes	IHC	CD1a and S-100 negative histiocytes.	Observation and treatment of lymphadenopathy and lytic bone lesions as presumed metastatic breast cancer (60 months, progressive disease) -> diagnosed with ECD based on bone biopsy, started PEG-Interferon (5 months, complete metabolic response) -> Sustained complete response for 144 months, although	Clinical, PERCIST, RECIST	Complete metabolic response	N/A	N/A

										patient now diagnosed with both DLBCL and recurrent breast cancer.				
7	Female	34	Yes	PET	Pituitary	Diffuse lymphadenopathy and lytic bone lesions	Yes	IHC	BRAF negative.	Diagnosed with diabetes insipidus, observation following DDAVP treatment (progressive disease, 144 months) -> Cytarabine and PO steroids (12 months, partial response).	PERCIST	Partial metabolic response	0.9, 13.3%	N/A
8	Male	40	Yes	MRI	Intracranial	None	Unknown	Unknown	CD68 and CD163 +, CD1a -. Associated astrocytic gliosis and minimal lymphocytic inflammation.	Seen for initial evaluation and then followed with local hematologist, no records available.	Unknown	Unknown	Unknown	Unknown
9	Male	49	Yes	CT	Retroperitoneal fibrosis with obstructive uropathy	Inflammatory pulmonary changes	Yes	IHC	BRAF negative.	Diagnosed following obstructive hydronephrosis with acute renal failure, Imatinib (1 month, stopped due to toxicity) -> Interferon (6 mon, unknown response) -> Vemurafenib (5 years, stable disease). Incomplete records following this.	Clinical, RECIST	Stable disease	0.4, 9.4%	N/A
10	Female	48	Yes	PET, CT, MRI	Bone	Spleen	Yes	IHC, NGS, FISH	BRAF V600E negative. Eosinophilia FISH panel negative. Cytogenetics 46, XX [20]. NRAS positive in the marrow and spleen. Myeloid NGS reveals a Tier II (variant of potential clinical significance) NRAS mutation, c.182A>G, p.Q61R, at a variant frequency of 5%.	Presented with lytic bone lesions, observation for 5 years until diagnosis of ECD made -> Started Cladribine, unknown response. Follows with local hematologist.	Unknown	Unknown	1.1, 14.9%	N/A
11	Male	52	Yes	CT	Bone	Retroperitoneal fibrosis, perivascular soft tissue involvement (aorta, IVC) and colonic involvement	Yes	NGS and cfDNA	BRAF V600E +	Diagnosed 2015, started Anakinra x1 year then switched to Vemurafenib after discovering BRAF mutational status -> Continued Vemurafenib (3 years, stable disease , self-discontinued for unknown reason x2 mon) -> Started pembrolizumab (5 month, stopped due to toxicity) -> Resumed Vemurafenib (11 months, stable disease).	RECIST	Stable disease	1.1, 8.6%	N/A
12	Male	45	Yes	PET	Liver	Retroperitoneal fibrosis	Yes	IHC and NGS	BRAF and KRAS staining negative. Omental and falciparum biopsy: CD163-positive histiocytes, positive for factor XIIIa, negative for S-100, langerin, CD1a.	Presented with elevated liver enzymes in 2021, diagnosed with autoimmune hepatitis, treated with steroids (13 months, progressive disease) -> Omental biopsy consistent with ECD -> Cobimetinib started (10 months, complete response).	Clinical, PERCIST	Complete response	0.9, 14.8%	0.5, 5.4%
13	Female	65	Yes	CT	Mesentery	Perivascular thickening of aorta and other large vessels	Yes	IHC	BRAF staining negative. Histologic changes in the omentum and perianal lesion. Histocytes positive for CD68, negative for S100. Few IgG4 positive plasma cells.	Presented in 2018 with enteritis, underwent biopsy in 2019 which was consistent with ECD. Diagnosed with vasculitis at the same time. -> Steroids and methotrexate started (12 months, progressive disease) -> Off therapy (4 months, progressive disease) -> Cobimetinib started (3 months, partial response) -> Cobimetinib dose increased (34 months, complete response) -> Cobimetinib stopped due to side effects (6 months, complete response off therapy).	Clinical, PERCIST	Complete response	1.2, 5.2%	N/A
14	Male	58	Yes	MRI	Orbits	Bone, soft tissue in mediastinum, encasement and thickening of adrenals	Yes	IHC	Weakly BRAF positive.	Presented in 2019 with bilateral orbital masses, biopsy consistent with ECD (2 months, progressive disease off therapy)-> Interferon alpha started (11 moths, mixed response) -> Trametinib started (9 months, stable disease) -> Trametinib stopped due to side effects; Vemurafenib started (2 months, progressive disease) -> Vemurafenib stopped due to side effects; Radiation to bilateral orbits (5 months, stable disease) -> Off therapy (4 months, progressive disease) -> Cladribine started (3 months, stable disease).	Clinical, RECIST	Stable disease	0.82, 6.5%	0.1, 0.4%
15	Male	53	Yes	CT	Renal	Retroperitoneal fibrosis, pericardial and	Yes	IHC	BRAF-positive staining.	Presenting with recurrent pyelonephritis, status post nephrectomy -> Progressive disease, 7 years) ->	Clinical, RECIST	Complete response	0.5, 5.6%	N/A

						epicardial fibrosis, bones				Vemurafenib started (4 months, progressive disease) -> Dabrafenib started (13 months, stable disease) -> Self discontinued Dabrafenib, off therapy (15 months, complete response).				
16	Female	54	Yes	CT	Pituitary (diabetes insipidus)	Breast, retroperitoneal fibrosis, periaortic thickening	Yes	IHC	N/A	Presented in 2013 with diabetes insipidus -> No therapy (36 months, progressive disease) -> 2016 biopsy of breast mass consistent with ECD -> Started Vemurafenib (Stable disease, 48 months).	Clinical, RECIST	Stable disease	0.7, 4.4%	N/A
17	Female	48	Yes	MRI	Brain (panhypopituitaris m)	Bilateral masses surrounding cervical vertebral arteries, soft tissue masses in chest wall, iliopsoas)	Yes	IHC	BRAF positive staining. Histiocytes positive for CD88, negative for S100, and PAX8. CD45 positive.	Symptom onset in 2006, diagnosed with panhypopituitarism in 2009, (Progressive disease, 12 years) -> Biopsy proven ECD in 2018; Vemurafenib started (discontinued after one month due to dysphagia and cognitive impairment) -> Off therapy, lost to follow-up.	Clinical, RECIST	Progressive disease	0.72, 4.5%	N/A
18	Male	78	Yes	CT, MRI	Bones	Unknown	Yes	IHC	BRAF staining negative. Osteosclerosis with histiocytes. positive for CD45 and CD68. Negative staining for pancytokeratins (AE1/AE3), S100, CD117, and CD34.	Presented with 5 years of knee pain, biopsy in 2019 consistent with ECD -> Lost to follow-up due to lack of social support and significant debility.	Clinical	Progressive disease	N/A	N/A
19	Male	61	Yes	PET, MRI	Facial bones	None	Yes	IHC	BRAF positive. Diffuse positivity for CD68. CD1A and S-100 are negative.	Presented with nasolabial mass in 2016, biopsy consistent with ECD. Previously on methotrexate and prednisone for rheumatoid arthritis, no therapy (12 months, progressive disease) -> Local radiation (progressive disease within one year) -> Local radiation (complete response, 7 years).	Clinical, PERCIST, RECIST	Complete response	0.3, 9.7%	N/A
20	Female	55	Yes	PET, MRI	Facial bones	Possible rib involvement	Yes	IHC	BRAF positive. Histiocytes positive for CD68 and negative for S100 and CD1a. Tryptase and CD117 stain scattered, round mast cells.	In 1995 had rib removed due to mass and pain, biopsy showed eosinophilic granulomatosis -> 2011 new onset bone pain with non-diagnostic bone marrow biopsy -> Observation for two years with new retro-orbital soft tissue hypertrophy. Biopsy consistent with ECD -> Interferon started (progressive disease, 2 months) -> Vemurafenib started, self-discontinued after three months, unknown response -> Methotrexate started by Rheumatology, no response assessment -> Vemurafenib started again 2015 -> (Stable disease, 8 years).	Clinical, PERCIST, RECIST	Stable disease	0.5, 5%	N/A
